# ‘Back and forth’ from models to patients to understand kidney disease: an interview with Katalin Susztak

**DOI:** 10.1242/dmm.018911

**Published:** 2014-12

## Abstract

Katalin Susztak is currently Associate Professor at Perelman School of Medicine at the University of Pennsylvania, where she conducts research on chronic and diabetic kidney disease. After her medical studies, her science career began by investigating ion-channel regulation and function at Semmelweis University, in Budapest. She then moved to the United States, where, thanks to the fortunate encounter with her future mentor, Erwin Böttinger, she worked as a research fellow in the Renal Division of the Albert Einstein College of Medicine in New York. Since then, she has successfully kept pursuing her career in the field of kidney research. The innovative approach of Katalin’s lab is to combine efficient high-throughput analysis of patient samples with mechanistic approaches in animal models, in order to advance our understanding of kidney disease mechanisms and identify new therapeutic targets. In this interview, Katalin travels through her career, from her first steps into biophysics, to her residency and finally to her established position as a kidney-specialist scientist, discussing exciting aspects of her work and current challenges in her field.

Katalin Susztak was born in Eger, Hungary, in 1971. She obtained her MD degree in 1995 from Semmelweis University Medical School in Budapest, where she stayed for her PhD studies. In that period, she worked at the Department of Physiology and conducted research on the role of a proton-conducting channel in the regulation of neutrophil function. She then moved to the United States, where she started her residency in medicine at the Albert Einstein College of Medicine in New York. Soon after starting her short-track program, she met her future mentor, Dr Erwin Böttinger, and went immediately back to the bench to study the interplay between Smad proteins and transforming growth factor-β, and to perform genome-wide gene expression studies, using the newly developed gene chip method. Since then, she has been investigating the molecular pathways that regulate chronic and diabetic kidney disease, by combining high-throughput genetic, genomic and epigenetic analyses on patient samples with mechanistic approaches in animal models. She is now Associate Professor at the University of Pennsylvania and has also recently joined the team of academic editors at *Disease Models & Mechanisms*.

**You started your journey into science as an electrophysiologist, studying ion-channel function and regulation. How did your interest in nephrology come about?**

Actually, it is a complicated story. Just after my PhD thesis, I moved to the US and immediately started working in the hospital. At the beginning, the transition from the research activity of the lab to the full-time immersion into medical procedures was very hard. However, just 2 months after I had started my residency, I had a patient who got paralyzed because of hypokalemia, low potassium in the blood. In that situation, I was able to use my knowledge to diagnose a rare disease, thanks to my expertise in ion channel physiology – an ion-transport problem had led to the development of hypokalemia in that patient. That was my first interaction with physicians where my scientific knowledge and rigorous methodical training helped to unravel a clinical case. I was particularly impressed by the consultant who followed this case. He was the head of the Renal Division and a physician scientist himself. I think he must have been as surprised as I was by him (finally a scientist!) finding an intern who knew about ion transport and ion-channel regulation. He immediately invited me to visit his lab in the Renal Division and there I met for the first time my future mentor, Dr Böttinger. He had just been recruited at the Albert Einstein College of Medicine and was working on the application of microarrays and high-throughput transcriptomic and genomic analyses in kidney disease. I fell in love with that lab straight away – it was ‘love at first sight’ for me – and I decided to stay with Dr Böttinger as a research fellow. That was a very exciting time in renal research, with new genetic techniques being explored. For me, it was also extremely useful training, as I started to do completely new things, moving from biophysics to genetics and gene regulation. Then everything went very fast: I finished residency, I did a year of clinical work to be certified in nephrology, and was then promoted to be an Instructor, and, step-by-step, I established my career in kidney research.

**Figure:**
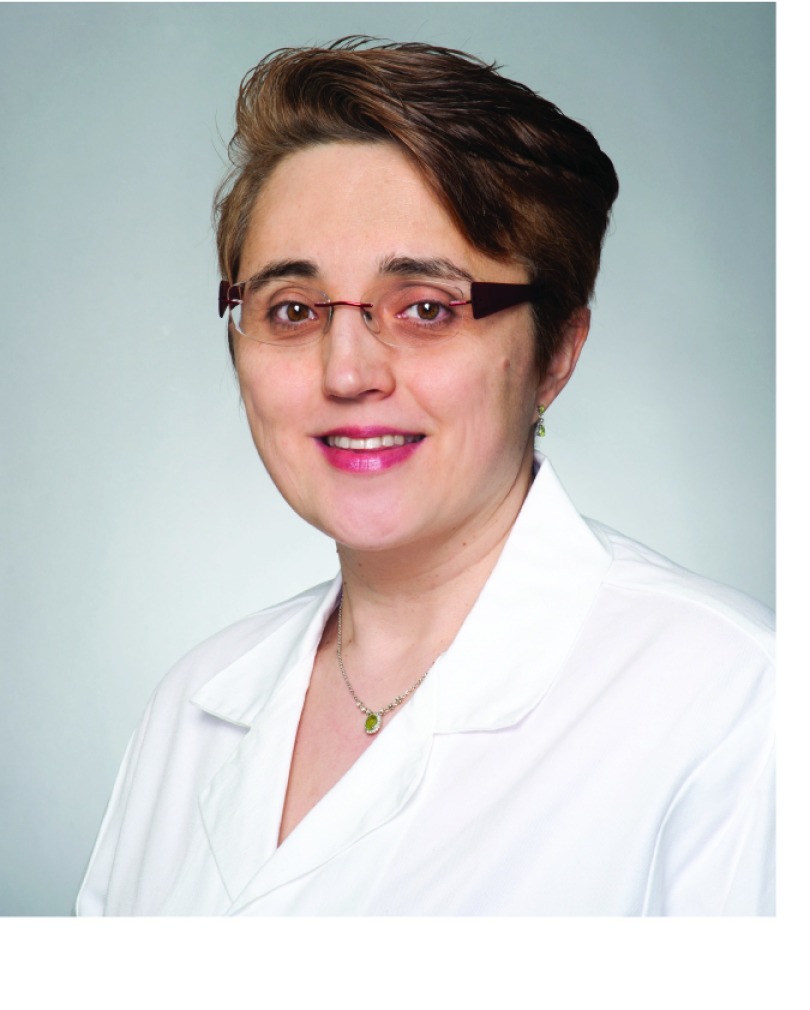

“That was a very exciting time in renal research, with new genetic techniques being explored. For me, it was also extremely useful training, as I started to do completely new things, moving from biophysics to genetics and gene regulation”

**Talking about the evolution of your research interests, you are now employing ‘omics’ approaches to directly screen patient samples. How did you move towards more translational research?**

After residency, when I was back to the lab full time, I decided to use my knowledge as a clinician and nephrologist to move from basic research to more translational approaches, in order to better understand chronic and diabetic kidney disease. During that period, my lab was part of a consortium to develop ‘human-like’ mouse models for kidney diseases. Soon I realized that, although models can faithfully recapitulate certain aspects of the human condition, they always remain ‘models’, and often we discover differences between mouse models and the human condition. I realized that it is very important to collect patient samples and compare our findings between mice and humans. So I started characterizing human samples from healthy and affected individuals, and for that project I received a Career Development Award, which represents an important step here in the US. Initially, we were able to analyze only samples from small cohorts of patients, but now [at the Perelman School of Medicine] we have one of the biggest kidney biobanks, with more than 1000 kidney samples. Of course, the type of analysis we perform has also evolved. Initially, we were interested in the transcriptome and were mainly doing microarrays to identify pathways that could be relevant for kidney disease development. But now we have a much more comprehensive and integrative approach, which combines epigenetics, genetics and transcriptomics, and we moved to using next-generation sequencing methods.

“I realized that, although models can faithfully recapitulate certain aspects of the human condition, they always remain ‘models’…it is very important to collect patient samples and compare our findings between mice and humans”

**In 2009 you got an NIH grant for the project called ‘Epigenetics Landscape of Chronic Kidney Disease’. Why did you work on epigenetics in particular?**

That project was funded as part of the so-called NIH Roadmap Epigenomics Consortium, whose aim was to characterize the epigenome in non-malignant diseases. There was in fact already at lot of knowledge about the role of epigenetics in different types of cancer, but we didn’t know much about epigenetic alterations in other diseases. Inside the consortium, we proposed to combine gene expression studies with epigenomic analysis to facilitate the identification of new diagnostic and prognostic markers for progressive renal disease. There were a lot of good collaborators in the consortium so we learnt a lot. Our project was funded originally until 2014, but we renewed it for another 5 years. Now we have different goals: initially, we wanted to compare epigenomic maps between control and diseased kidney samples to identify any relevant differences; now we would like to use the kidney-specific epigenome maps to find driver pathways for kidney disease development as well as gene and gene-regulatory alterations, so that we can understand the function of polymorphisms previously identified in genome-wide association studies [GWAS].

**Could you please explain the route from patient samples to new therapeutics? How can we use the knowledge acquired from the study of those samples to develop new therapeutic strategies?**

Actually, there are several ways to do that, but we need both human samples and animal models. First, we can use the knowledge obtained using high-throughput genetic and genomic methods to screen human samples to identify candidate pathways that might be useful therapeutic targets. For example, using such studies we found that development-related molecular pathways, such as Notch, are activated in kidney disease samples. As Notch is involved in regeneration and stem cell functions, we thought that this pathway could have a beneficial effect in kidney disease, maybe driving some processes of healing in response to kidney damage. But, actually, we found that the activation of this pathway caused more damage during chronic kidney disease in mouse models. Instead, if we inhibited Notch signaling in tubular epithelial cells in the kidney, this significantly ameliorated the disease, which suggests that the inhibition of Notch might be potentially therapeutic.

Then there are other approaches. An area we are also interested in is translating many of the genetic signals obtained through GWAS into disease-targeting strategies. These association studies have highlighted multiple polymorphic sites that are associated with chronic kidney disease and diabetic nephropathy development. Most of these sites are in the non-coding regions of the genome, so we don’t know what their function is and how they can contribute to disease development. Our collection of samples is very useful in this context, as we can map these regions, identify those polymorphisms that are in key regulatory regions of kidney-disease-associated genes, and define which genes could be potential targets of the genetic polymorphisms. Once we have identified potential target genes, we can do more functional analyses in model organisms. We mostly work with mice but have now started to work with zebrafish, because it is a very nice high-throughput system.

**Of course, model organisms are fundamental to perform functional analyses and test experimental hypotheses. But how good are current models of kidney disease in mimicking the human condition?**

Well, unfortunately, mice are not an ideal model for kidney disease: although they do develop certain features of both chronic kidney disease and diabetic nephropathy, they don’t develop the full spectrum of the disease. We have spent 10 years in the Animal Models of Diabetic Complications Consortium [AMDCC] trying to identify the best mouse model for diabetic kidney disease. We have characterized a large number of different models and compared them to patient samples, but none of them fully recapitulate the complexity of the human condition. I think we really need to characterize these models in depth. Thanks to novel high-throughput automated systems, we can now do these comparisons nicely and efficiently. This comparative approach will allow us to identify the similarities and differences in gene expression between animal and human samples, and then use mice to study only those pathways that are similar to humans, but not those that are differentially regulated between the two species.

**What can the zebrafish model offer in this respect?**

Zebrafish is extremely useful in terms of looking into basic mechanisms, contributions of specific genes, or defining connections between gene regulators and targets. For example, we use zebrafish to identify genetic regions with kidney-specific regulatory activities that are present in the human genome, so we can clone those regions into a reporter and then look at their expression and function in zebrafish. Some of this work can also be done in mice but, because of the longer gestational period and higher costs in mice, zebrafish represent a time- and cost-effective model. Zebrafish is also ideal as a first screening tool for therapeutics, since it is possible to mimic some features of chronic kidney disease in this organism.

“Once we find new targets, we can use animal models to provide proof-of-principle results and test causality between a specific target and a disease feature, and then go back and forth between animal and human samples to make this process more efficacious”

**Which is the biggest challenge in kidney research at the moment?**

One of the biggest issues in our field is that current treatments for kidney disease are limited. This is particularly true for diabetic kidney disease. The incidence of diabetes will be one in three people in a couple of years, so it’s going to be a very common condition, and, actually, since insulin was discovered, acute hyperglycemia hasn’t been the leading cause of mortality; instead, this is now more associated with diabetes complications, such as cardiovascular and renal complications, with the latter being the most common. That’s why the NIH funded a consortium to study complications of diabetes in mouse models to better understand mechanisms of diabetic kidney disease and develop new therapeutics. Very few studies involving novel therapeutics have been initiated in the last decade, so we need new treatments, which means that we really need to find new potential targets. Once we find new targets, we can use animal models to provide proof-of-principle results and test causality between a specific target and a disease feature, and then go back and forth between animal and human samples to make this process more efficacious.

**How far are we from the development of new drugs?**

New compounds are developed all the time but very few reach final approval, even considering the great potential of the repurposing of existing drugs from other fields to nephrology. What prevents new drugs from reaching the end-stage point in the drug discovery pipeline is the lack of patient-derived samples for specific disease subtypes that can be used in pre-drug screenings. For example, oncologists initiated the Cancer Genome Atlas Project, in which patient-derived samples are used to identify specific changes associated with cancer subtypes and use this knowledge to improve prevention, early detection and treatment for cancer. We are also working on something similar in the nephrology field, but I think we would need a much bigger and concentrated effort to define proper disease subtypes of chronic and diabetic kidney disease. Those are very heterogeneous diseases for which it would be ideal to have personalized treatments. This I think should be the next big step in nephrology research.

“I think we would need a much bigger and concentrated effort to define proper disease subtypes of chronic and diabetic kidney disease. Those are very heterogeneous diseases for which it would be ideal to have personalized treatments. This I think should be the next big step in nephrology research”

**Do you have any recommendations for young scientists?**

I think this is a really exciting time in science. New technologies are emerging, which will really accelerate research progress, and I think we have fantastic new discoveries ahead of us in biology. The approach to science is likely going to be different. We need team-based research projects to use more approaches at the same time in order to understand disease mechanisms and search for new treatments. And, I think especially for young researchers, it will be very important to broaden their background as much as possible, learning physiology, genetics, but also gaining computational skills to extract useful information from the huge amount of data being collected.

“I think this is a really exciting time in science. New technologies are emerging, which will really accelerate research progress, and I think we have fantastic new discoveries ahead of us in biology”

**If not science, what would you do?**

I am now equally attracted by medicine and science. Even though I spend only 20% of my time in the hospital, I really enjoy working with patients. So, if I were about to leave science, I would most likely be a full-time clinician.

**How do you enjoy your free time out of the lab and the hospital?**

I like being with my family. My kids are 10 and 14. They are a great source of joy: it’s great to talk to them and learn about their ideas, and to watch them growing and evolving. And then I really love cooking, and, as I’m not doing experiments anymore, my kitchen has replaced the lab. And, fortunately for me and my family, most of my ‘kitchen’ experiments turn out to be successful!

